# Experimental Study of Damage Evolution in Circular Stirrup-Confined Concrete

**DOI:** 10.3390/ma9040278

**Published:** 2016-04-08

**Authors:** Zuohua Li, Zhihan Peng, Jun Teng, Ying Wang

**Affiliations:** 1 School of Civil and Environment Engineering, Shenzhen Graduate School, Harbin Institute of Technology, Shenzhen 518055, China; lizuohua@hit.edu.cn (Z.L.); hayespeng@gmail.com (Z.P.); 2 IoT Application Technology Center of NDT, Shenzhen Graduate School, Harbin Institute of Technology, Shenzhen 518055, China; 3 Department of Civil and Environmental Engineering, University of Surrey, Guildford GU2 7XH, UK

**Keywords:** concrete, confinement, circular stirrups, damage evolution, experimental study

## Abstract

This paper presents an experimental study on circular stirrup-confined concrete specimens under uniaxial and monotonic load. The effects of stirrup volume ratio, stirrup yield strength and concrete strength on damage evolution of stirrup-confined concrete were investigated. The experimental results showed that the strength and ductility of concrete are improved by appropriate arrangement of the stirrup confinement. Firstly, the concrete damage evolution can be relatively restrained with the increase of the stirrup volume ratio. Secondly, higher stirrup yield strength usually causes larger confining pressures and slower concrete damage evolution. In contrast, higher concrete strength leads to higher brittleness, which accelerates the concrete damage evolution. A plastic strain expression is obtained through curve fitting, and a damage evolution equation for circular stirrup-confined concrete is proposed by introducing a confinement factor (*C*) based on the experimental data. The comparison results demonstrate that the proposed damage evolution model can accurately describe the experimental results.

## 1. Introduction

In engineering applications, the allocation of reinforcement stirrups is an important measure to improve the mechanical properties of compression members or other structural components under compression because stirrups allocated perpendicularly to the axial compression/maximum principal stress orientation are able to confine the transverse deformation of the core concrete. Stirrup-confined concrete have been studied since 1903, when Considère and Moisseiff [1] first indicated that transverse stirrups improved the deformation capability of axial compression columns. Over the past century, numerous researchers have conducted theoretical and experimental studies on confined concrete including damage related functions and numerical models, *etc.* by using different approaches (Richart *et al.*, 1928 [2]; Kent and Park, 1971 [3]; Sheikh and Uzumeri, 1980 [4]; Mander *et al.*, 1988 [5]; Karabinis and Kiousis, 1994 [6]; Spoelstra and Monti, 1999 [7]; Montoya *et al.*, 2004 [8]; Papanikolaou and Kappos, 2007 [9]; Rousakis *et al.*, 2008 [10]; Karabinis *et al.*, 2008 [11]; Monti and Nisticò, 2008 [12]; Moghaddam *et al.*, 2010 [13]; Jiang and Wu, 2012 [14]; Peter *et al.*, 2013 [15]; Nisticò and Monti, 2013 [16]; Nisticò *et al.*, 2014 [17]; Gambarelli *et al.*, 2014 [18]; Nisticò, 2014 [19]; Wei and Wu, 2014 [20]). These studies confirmed that confinement (steel and/or FRP) improved the strength of reinforced concrete members. Particularly for the high-strength concrete with stirrup confinement, the strength increased much higher than that of unconfined concrete [21]. Not only improving the concrete strength, stirrups can lead to the increase of the ultimate compressive strain so the ductility is improved as well [5,13]. In order to quantify these effects, various confined concrete stress-strain models have been proposed by numerous researchers (Kent and Park, 1971 [3]; Sheikh and Uzumeri, 1982 [22]; Mander *et al.*, 1988 [23]; Saatcioglu and Razvi, 1992 [24]; Cusson and Paultre, 1995 [25]; Moghaddam *et al.*, 2010 [26]; Samani and Attard, 2012 [27]).

Concrete is a multiphase composite quasi-brittle material, and its damage mechanism is complicated. The cracking process of concrete is different from those of other brittle materials. Research indicated that when maximum principal stress reaches a certain level, a large number of microcracks may exist in concrete, especially at the interface between coarse aggregates and mortar [28,29]. Concrete cracking process, which can be studied at a microscopic or even macroscopic level [30], is a continuous formation and merging of microcracks. Such a process eventually leads to the concentration of multiple microcracks in a very narrow area, and then a visible macrocrack forms, which causes the cross-section stiffness degradation [31,32]. Numerous modern measurement techniques have been applied to the research on concrete damage mechanism at a microscopic level, with reliable results [33,34,35,36,37,38,39,40], but sophisticated equipment is required. Numerical simulation is another option. However, because of the complexity of numerical algorithms and the computational cost, efforts on the simulation of the detailed evolution (growth and coalescence) of each microcrack in stirrup-confined concrete are inefficient. Alternatively, the phenomenological approach at the macroscopic level could be used to describe the effects of microcracks on the damage evolution. Macroscopic phenomenology studies of concrete damage mechanism have obtained excellent achievements, which improved the understanding of concrete damage evolution by measuring the variation of material properties (e.g., elastic modulus and compression strength) [41,42,43,44,45], whereas most of these studies concentrated on unconfined concrete and the research on damage evolution of stirrup-confined concrete has not been systematically conducted yet.

This paper aims to investigate the damage evolution in circular stirrup-confined concrete specimens through experimental study. The tangent module degradation was defined as a damage indicator, and it was subsequently used to obtain damage evolution curves. Furthermore, the effects of stirrup volume ratio, stirrup yield strength and concrete strength on damage evolution of the stirrup-confined concrete are discussed. To describe the effects of various confinement parameters on concrete damage evolution, a damage evolution equation is proposed by introducing a confinement factor (*C*). The proposed damage evolution equation can represent the experimental results reasonably.

## 2. Specimen Tests

### 2.1. Materials

Three groups of concrete with different strengths named as groups C1, C2 and C3 were prepared for concrete specimens. The material compositions of those concrete are listed in Table 1. The compressive strength of 150 mm × 150 mm × 150 mm concrete cubes were measured according to the Chinese Standard GB/T 50081-2002 [46]. The 28-day cubic compressive strength of each group is listed in Table 2. Standard deviations of three test results are 0.66, 0.91 and 1.9, respectively, much lower than the standard requirement [47]. Two groups of stirrups named Y1 and Y2 with different yield strengths were used, with diameters of 6.0 and 6.5 mm, respectively. The diameter of the longitudinal steel bars was 10.0 mm. Tensile tests were performed to measure yield strength and ultimate tensile strength of stirrups and steel bars, and the results are listed in Table 3. The standard deviations of three test results are much lower than the standard requirement.

### 2.2. Specimen Design

Thirty standard circular concrete specimens were manufactured with the same dimensions of Φ 150 mm × 300 mm. Among them, eighteen were stirrup-confined concrete specimens, while the other twelve were unconfined concrete specimens. The variables in the tests were concrete compressive strength, stirrup volume ratio and stirrup yield strength. In this study, the compressive strengths of groups A, B, and C concrete are 21.3, 25.5 and 40.5 MPa, respectively. Based on practical engineering experiences, three stirrup volume ratios were chosen as 0.92%, 1.84% and 2.75%, denoted as S1–S3, respectively. Y1 and Y2 denote stirrup yield strengths are 370 and 536 MPa, respectively. S0Y0 denotes the unconfined concrete. The mechanical and geometrical properties of all specimens are listed in Table 4, where λ*_v_* is stirrup characteristic value [48] and ω*_wd_* is mechanical volumetric ratio [49], which are given by:

(1) $$\lambda_{v}=\frac{\rho_{v}f_{y}}{f_{cu}}$$

(2) $$\omega_{wd}=\frac{\text{volume of confining hoops}}{\text{volume of concrete core}}\times \frac{f_{y}}{f_{cu}}$$

**Figure 1:**
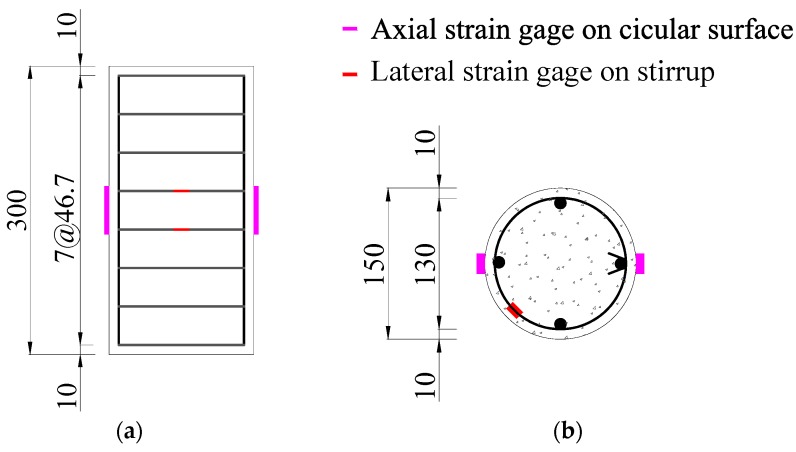
Construction drawing of specimen C2S3Y1 and locations of axial strain gauges and lateral strain gauges: (**a**) horizontal view; and (**b**) top view (unit: mm).

For ρ*_v_* is stirrup volume ratio, λ*_v_* and ω*_wd_* has the same meaning with different minimum required values. The minimum ω*_wd_* is 0.12 for column critical region at the base or 0.08 for column critical region above the base [49]. Accordingly, λ*_v_* have the minimum required values in range of 0.05–0.24 for columns with different anti-seismic grades, stirrup types and axial compression ratios [48]. It seems that the minimum ω*_wd_* is in the middle range of the minimum λ*_v_*. It is suitable and safe for common structural design. The construction drawing of a representative specimen C2S3Y1 with a stirrup spacing of 46.7 mm is shown in Figure 1 as an example.

### 2.3. Test Procedure

**Figure 2:**
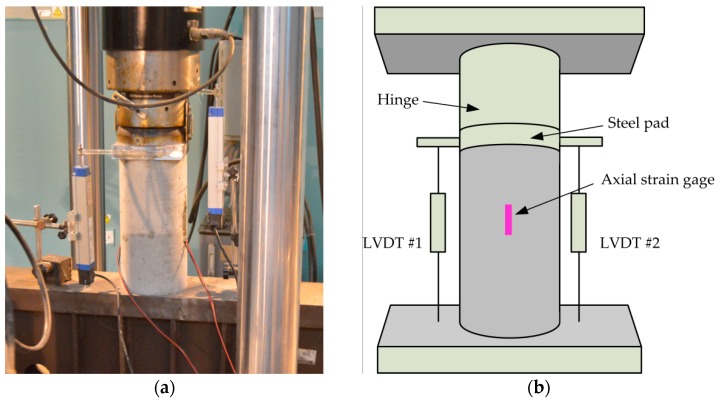
Test setup and instrumentation: (**a**) test setup; and (**b**) locations of strain gages and LVDTs.

The loading tests were performed using a 1000 kN MTS electro-hydraulic servo system; the overall view of test setup is shown in Figure 2a. Two 50-mm axial strain gages were attached on the surface of specimens at mid-height. The axial deformations of specimens were measured by two LVDTs. The locations of axial strain gages and LVDTs are shown in Figure 2b. The loading of the tests was displacement controlled with a loading rate of 0.4 × 10^−4^ s^−1^.

In this study, monotonic and cyclic loading compression tests were performed on all the specimens. Prior to the tests, the specimens were pre-loaded to 10% of the estimated peak load and then held for 60 s to eliminate the slackness of test system and avoid the eccentricity of loading by adjusting specimen position until strain gage readings were consistent.

The cyclic loading compression tests were performed on both unconfined and stirrup-confined concrete specimens. A constant strain increment (Δε) was used in each test. Because of the discrepancy in strain between specimens, two loading conditions with different strain increments were selected in the tests. For unconfined concrete, the magnitudes of strain increment (Δε) in two loading conditions were 5.0 × 10^−4^ and 7.5 × 10^−4^, whereas for stirrup-confined concrete specimens, the magnitudes were 1.00 × 10^−3^ and 1.25 × 10^−3^ or 0.50 × 10^−3^ and 1.00 × 10^−3^. Loading rate was 4 × 10^−5^/s for all specimens.

## 3. Test Results

### 3.1. Monotonic Loading Tests

**Figure 3:**
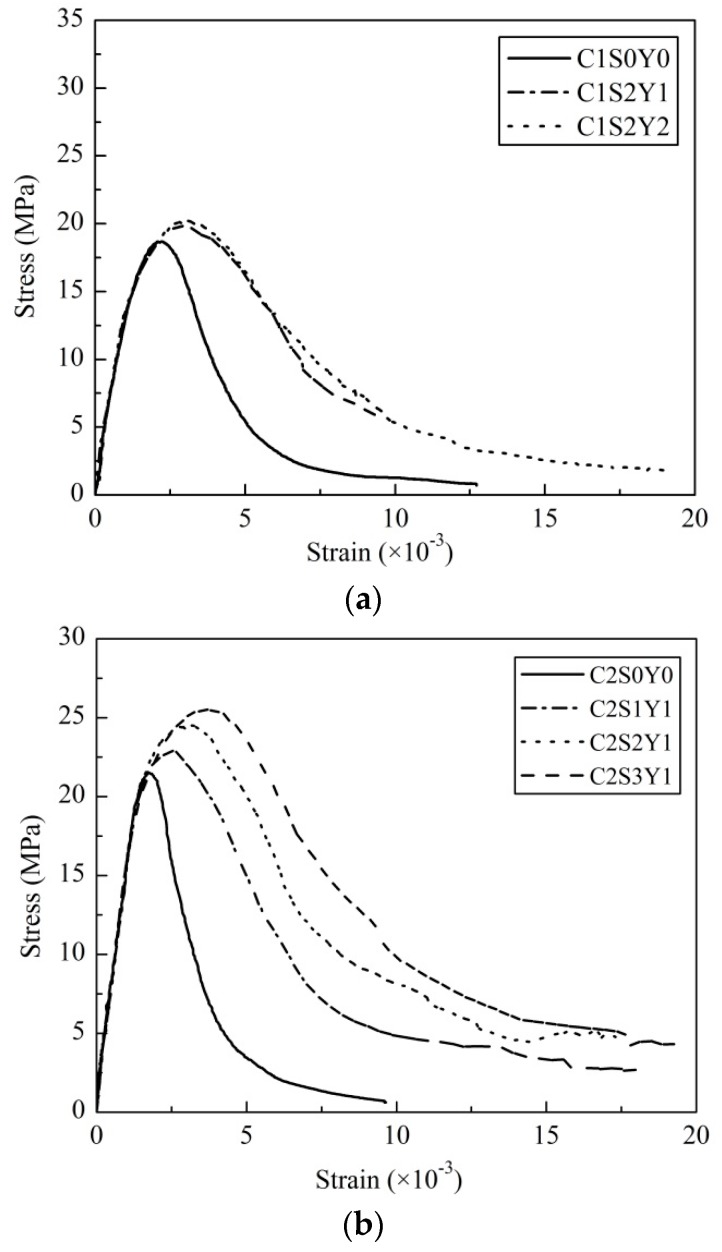

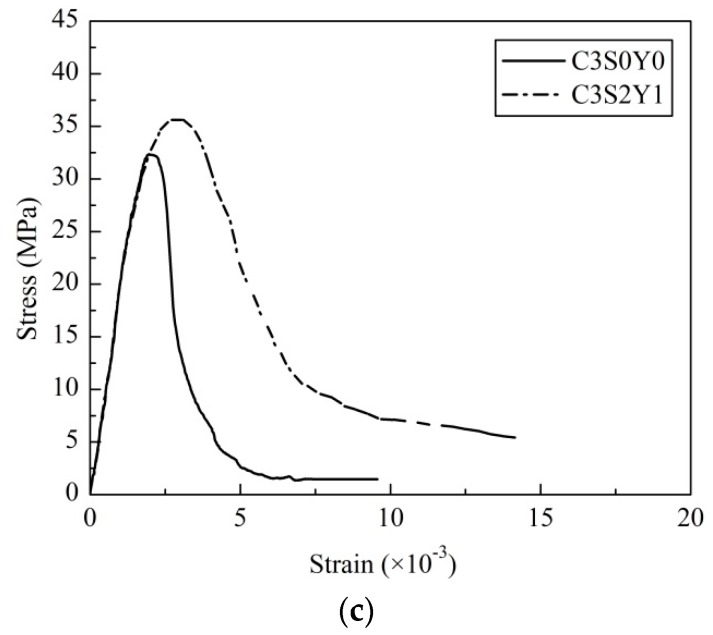
Monotonic stress-strain curves for: (**a**) Group C1 (*f_cu_* = 21.3 MPa); (**b**) Group C2 (*f_cu_* = 25.5 MPa); and (**c**) Group C3 (*f_cu_* = 40.5 MPa).

The monotonic stress-strain curves of all specimens are shown in Figure 3. Compared with unconfined concrete specimens, the stirrup-confined concrete specimens had visibly higher stress-strain peaks. Additionally, the ascending curves of stirrup-confined concrete specimens show a linear portion followed by a plateau and the descending curves were considerably broader with greater ductility. The stirrups yielded around the peak stress, at which the confinement effects were greatest.

### 3.2. Cyclic Loading Tests

**Figure 4:**
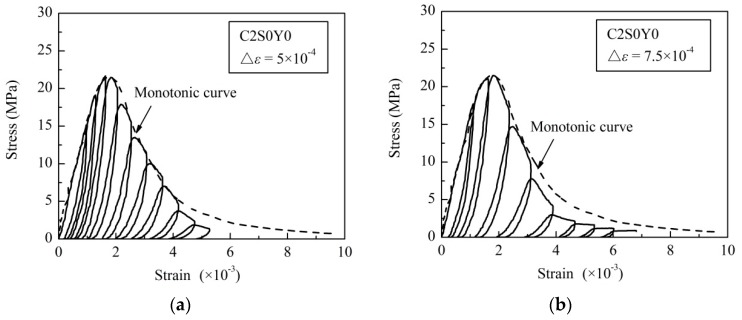

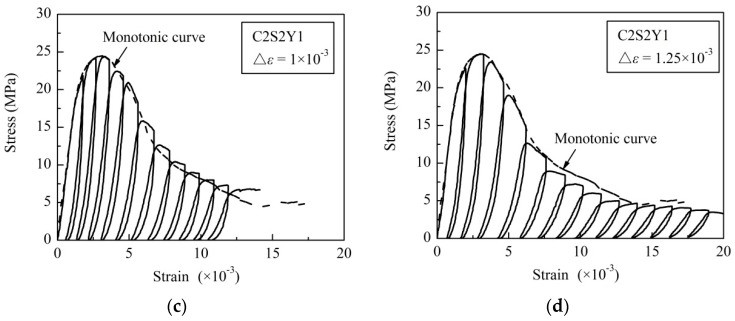
Cyclic stress–strain curves for: (**a**) unconfined concrete specimen (C2S0Y0) Δε = 5.0 × 10^−4^; (**b**) unconfined concrete specimen (C2S0Y0) Δε = 7.5 × 10^−4^; (**c**) stirrup-confined concrete specimen (C2S2Y1) Δε = 1.00 × 10^−3^; and (**d**) stirrup-confined concrete specimen (C2S2Y1) Δε = 1.25 × 10^−3^.

Figure 4 shows the stress-strain curves of two typical specimens under cyclic loading. The curves of unconfined concrete specimen C2S0Y0 are shown in Figure 4a,b, and those of stirrup-confined specimen C2S2Y1 are shown in Figure 4c,d. The corresponding monotonic stress-strain curves are also shown in the same figures. The envelope curves of cyclic loading tests present similar behavior with monotonic loading tests.

In the cyclic stress-strain curves, three characteristic behaviors can be observed:

1. When the load reached a certain level, complete unloading always led to plastic deformation. The strain of unloading points increased with the loading/unloading process progressed.
2. The curves showed obvious stiffness degradation during unloading process. With loading cycles increased, the unloading elastic modulus decreased continuously.
3. The stirrup-confined concrete bore more loading/unloading cycles with higher ultimate strain compared with the unconfined concrete when failure occurred.

**Figure A1:**
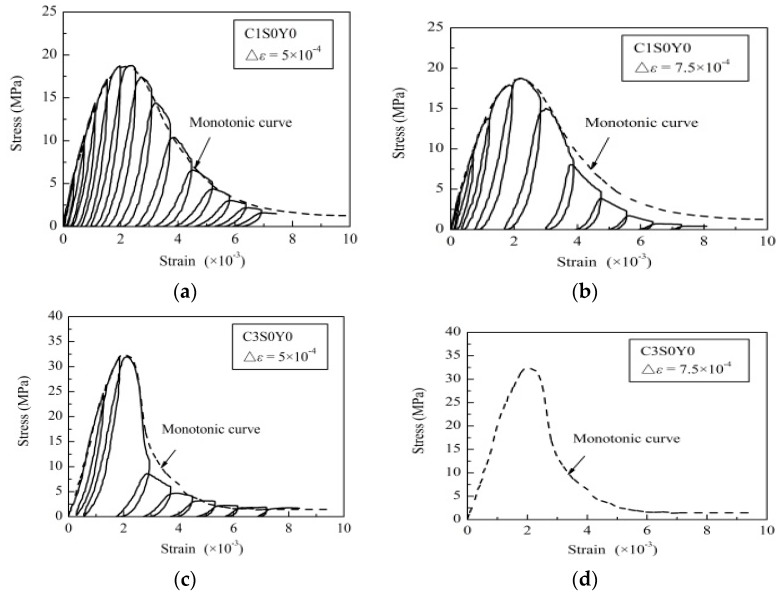
Cyclic stress–strain curves of unconfined concrete specimen: (**a**) C1S0Y0, Δε = 5.0 × 10^−4^; (**b**) C1S0Y0, Δε = 7.5 × 10^−4^; (**c**) C3S0Y0, Δε = 5.0 × 10^−4^; and (**d**) C3S0Y0, Δε =7.5 × 10^−4^.

**Figure A2:**
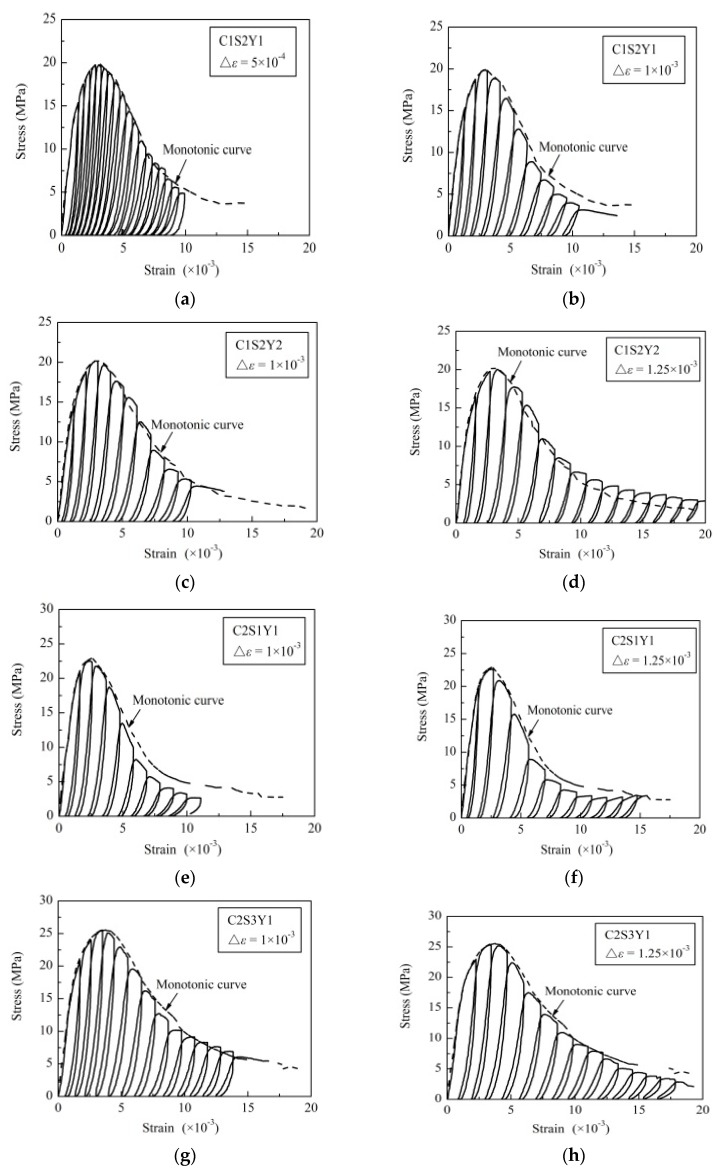

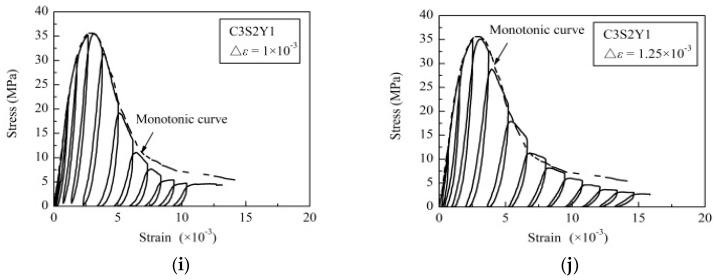
Cyclic stress-strain curves of stirrup-confined concrete specimen: (**a**) C1S2Y1, Δε = 5.00 × 10^−4^; (**b**) C1S2Y1, Δε = 1.00 × 10^−^^3^; (**c**) C1S2Y2, Δε = 1.00 × 10^−^^3^; (**d**) C1S2Y2, Δε = 1.25 × 10^−^^3^; (**e**) C2S1Y1, Δε = 1.00 × 10^−^^3^; (**f**) C2S1Y1, Δε = 1.25 × 10^−^^3^; (**g**) C2S3Y1, Δε = 1.00 × 10^−^^3^; (**h**) C2S3Y1, Δε = 1.25 × 10^−^^3^; (**i**) C3S2Y1, Δε = 1.00 × 10^−^^3^; and (**j**) C3S2Y1, Δε = 1.25 × 10^−^^3^.

In this study, two typical cyclic loading curves were selected for discussion. Curves of the remaining 14 specimens showed similar features, shown in Appendix (Figure A1 and Figure A2).

### 3.3. Failure Modes

**Figure 5:**
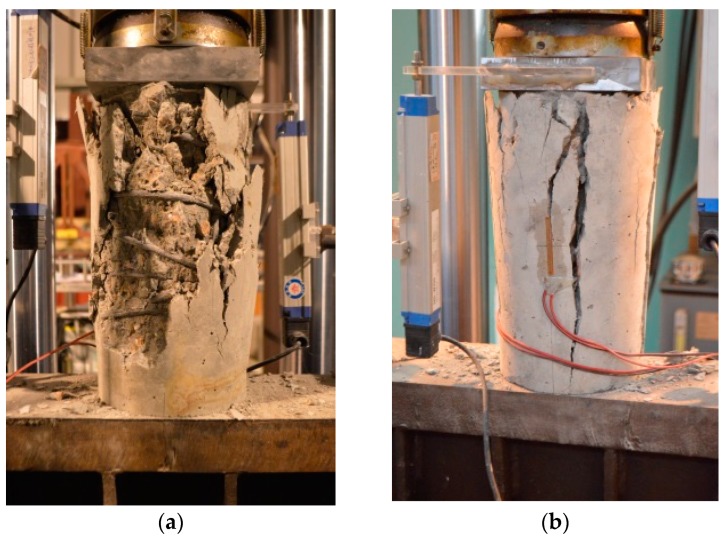
Failure patterns: (**a**) stirrup-confined concrete specimen (C2S3Y1); and (**b**) unconfined concrete specimen (C1S0Y0).

Figure 5 shows the failure modes under monotonic loading of typical stirrup-confined concrete specimen C2S3Y1 and unconfined concrete specimen C1S0Y0, respectively. For the stirrup-confined concrete specimen C2S3Y1, the initial longitudinal microcracks were formed parallel to the axial direction. With the increase of load, longitudinal microcracks progressed from both ends toward the center and merged with other longitudinal microcracks. Failure occurred after buckling of the longitudinal steel bars, and was explosive with total loss in load bearing capacity during stirrup relative slips. The stirrups bowed out due to the expansion of core concrete with upper end failure, as shown in Figure 5a. The stirrup slips made anti-buckling detailing failed, causing the longitudinal steel bars buckling. It suggested that the detailing preventing buckling and anchorage failure were important for confined concrete compression performance [50]. In contrast, for unconfined concrete specimen C2S0Y0, independent microcracks progressed, producing a major longitudinal macrocrack through the cross section, as shown in Figure 5b. It showed that load-bearing capacity reduced more rapidly after peak load and that the failure was more sudden and explosive.

## 4. Analysis and Discussions

### 4.1. Strength and Ductility of Stirrup-Confined Concrete

The test results for all specimens are listed in Table 5. The average peak compressive strain (ε*_c_*_0_) for three groups of unconfined concrete specimens was evaluated as 1.94 × 10^−3^, while those of the stirrup-confined concrete (ε*_cc_*) were in the range of 2.56 × 10^−3^–3.76 × 10^−3^. The ultimate compressive strain was defined as the strain when stress softened to 50% peak compressive stress. The average ultimate compressive strain (ε*_cu_*) of three groups of unconfined concrete was 3.33 × 10^−3^, while those of the stirrup-confined concrete (ε*_ccu_*) were in the range of 5.59 × 10^−3^–8.76× 10^−3^. The peak compressive stress ratios (*f*_cc_/*f_c_*_0_) and the corresponding peak compressive strain ratios (ε*_cc_*/ε*_c_*_0_) between stirrup-confined and unconfined concrete specimens are all greater than 1 for all specimens. The values of *f_cc_*/*f_c_*_0_ were in range of 1.07–1.19. Compared with the concrete strength improvement, the peak strain showed a more remarkable enhancement; ε*_cc_*/ε*_c_*_0_ were in the range of 1.43–2.12. In addition, the ultimate compressive strain ratios (ε*_ccu_*/ε*_cu_*) were in the range of 1.70–2.79. It clearly indicated the stirrup confinement improved concrete ductility.

Table 6 compares the prediction performances using typical confined concrete models with the values involving peak compressive stress ratio (*f_cc_/f_c_*_0_) and corresponding peak compressive strain ratio (ε*_cc_*/ε*_c_*_0_). In the table, for Kent and Park model [3], the underestimation of ε*_cc_*/ε*_c_*_0_ may be caused by using the same expression to estimate enhancements in both strength and strain, which is relatively approximate. The model proposed by Saatcioglu and Razvi [24] appears to give the estimable prediction of circular stirrup-confined concrete compared with the test results. For the theoretical model of Mander *et al.* [23], the test results show a difference with the prediction, which may be caused by the lower confinement effect because the specimen size limits the confinement densification detailing at the specimen end.

### 4.2. Damage Evolution of Stirrup-Confined Concrete

#### 4.2.1. Plastic Strain

The cyclic loading tests in this study showed that after a certain load limit was reached, the complete unloading of each loading cycle generated irreversible residual deformation, known as plastic deformation [51,52]. The strain of unloading point increased with the loading/unloading cycle continued, leading to higher levels of deformation, as shown in Figure 4.

By applying a quadratic polynomial expression to the ε*_p_*-ε relationship experimentally obtained, two functions were obtained for unconfined and stirrup-confined concrete, respectively. For simplicity, a unified function was proposed for both stirrup-confined and unconfined concrete specimens.

(3) $$\varepsilon_{p}=\left\{ \begin{array}{l} 0\;\;\;\;\;\;\;\;\;\forall \varepsilon \leq \varepsilon_{ce}\\ c{\left(\varepsilon -\varepsilon_{ce}\right)}^{2}+d(\varepsilon -\varepsilon_{ce})\;\forall \varepsilon >\varepsilon_{ce} \end{array}\right.$$

**Figure 6:**
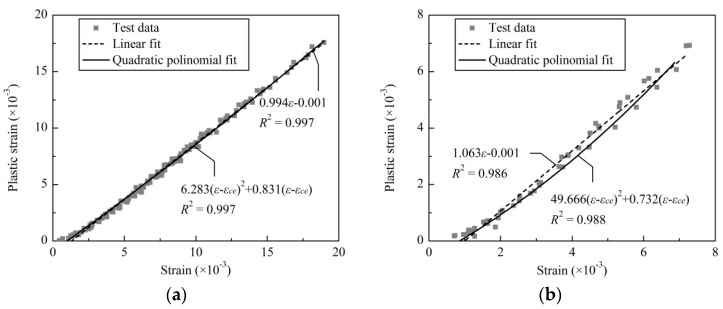
Proposed function for the ε*_p_*-ε relationship: (**a**) stirrup-confined concrete; and (**b**) unconfined concrete.

For comparison, a linear function is fitted, and the results are plotted together with the test data shown in Figure 6. The plastic strain curve of confined concrete shows more elastoplastic behavior. However, for unconfined concrete, it shows that the curve of linear fitting is an upper bound in intermediate strain range of 2.0 × 10^−3^–5.0 × 10^−3^, and quadratic polynomial fitting is close to an average curve in that range. With parameters *c* = 6.283 and *d* = 0.831, the proposed function of stirrup-confined concrete shows good correlation with the experimental data with *R*^2^ = 0.997, as shown in Figure 6a. Similarly, the parameters of unconfined concrete were obtained and a good correlation of *R*^2^ = 0.988 is observed, as shown in Figure 6b.

#### 4.2.2. Effects on Concrete Damage Evolution

The cyclic loading tests clarify that the concrete stiffness degrades because of the development of microcracks with the increase of plastic strain. In order to describe this phenomenon, a damage indicator *D* was defined to represent the evolution of concrete damage by Lemaitre [53]. According to this concept, the damage indicator *D* can be calculated by means of Equation (4), where *E*_0_ is initial elastic modulus and *E_u_* is unloading secant stiffness of the line connecting present unloading point to next loading point in the loading cycles.

(4) $$D=1-E_{u}/E_{0}$$

**Figure 7:**
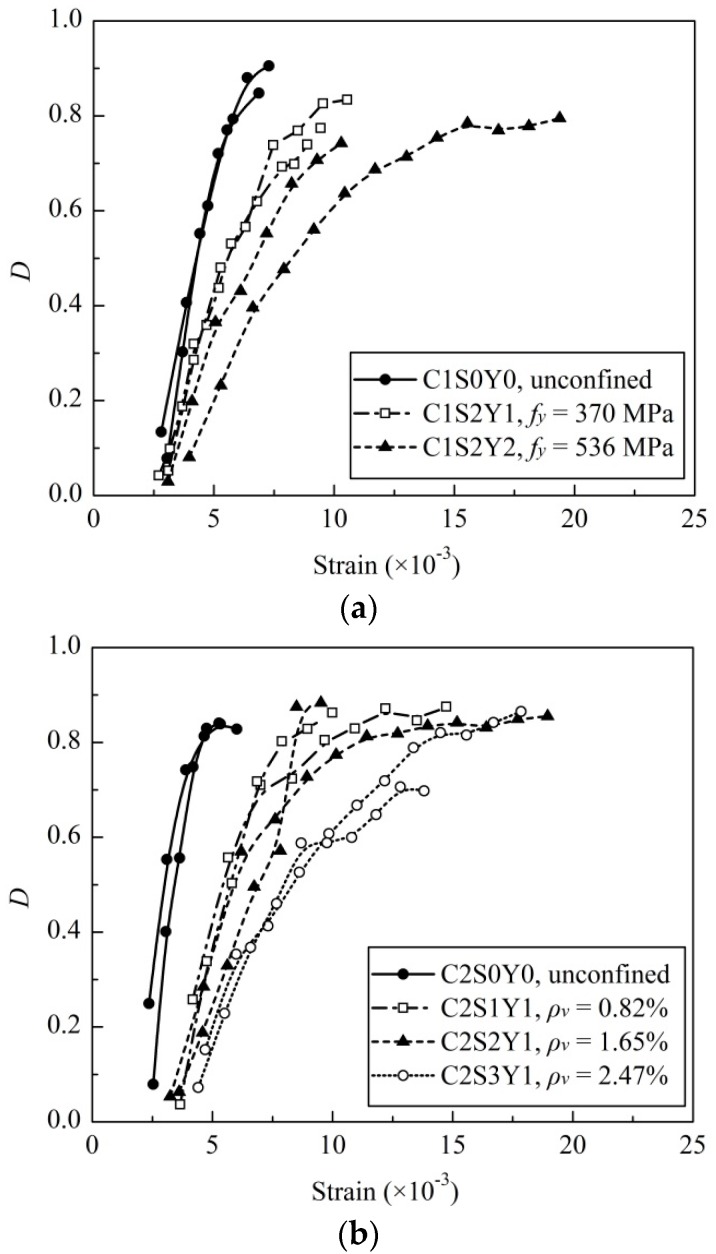

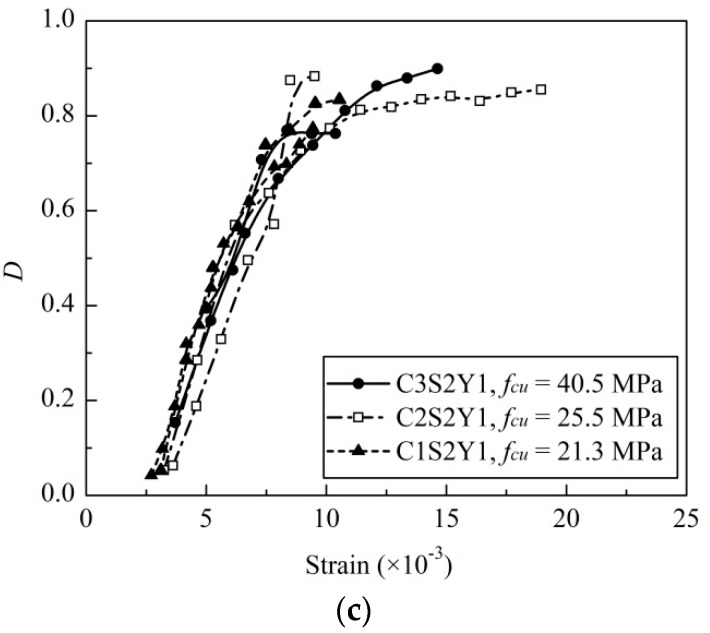
(**a**) Effect of stirrup volume ratio on damage evolution; (**b**) effect of stirrup yield strength on damage evolution; and (**c**) effect of concrete strength on damage evolution.

The effects of the stirrup volume ratio, stirrup yield strength and concrete strength on concrete damage evolution were investigated by comparing the test results, which are illustrated in Figure 7.

Figure 7a shows the effect of stirrup yield strength on the damage evolution of specimens with identical concrete strength and stirrup volume ratio, 21.3 MPa and 1.65%, respectively. It can be observed that the damage evolution of specimens with 536 MPa stirrup yield strength proceeded more slowly than those of specimens with 370 MPa stirrup yield strength. It demonstrates that the stirrup confinement effect is affected by stirrup yield strength to some extent. Higher stirrup yield strength leads to a higher confining pressure while inhibiting concrete damage evolution.

Figure 7b shows the effect of stirrup volume ratio on the damage evolution of specimens with identical concrete and strengths stirrup yield strengths, 25.5 and 370 MPa, respectively. For unconfined concrete specimens, damage emerged at ε = 2.0 × 10^−3^ and the curves increased sharply, suggesting rapid damage evolution. With stirrups allocated, damage emerged at strain in the range of 2.0 × 10^−3^–4.0 × 10^−3^, indicating that the damage evolution was delayed significantly. As compared with unconfined concrete, the damage evolution curve was gradual and longer. It is also observed in Figure 7b that the slopes of the curves gradually decreased, and that the ultimate compressive strain increased, as the stirrup volume ratio increased from 0.82% to 2.47%. It clearly demonstrates that when the stirrup volume ratio increased, the concrete damage evolution was restrained.

Figure 7c shows the effect of concrete strength on the damage evolution of specimens with identical stirrup volume ratios and yield strengths, 1.65% and 370 MPa, respectively. The high-strength concrete damage evolved more rapidly than low-strength concrete. The reason is that high-strength concrete exhibited limited transverse deformation under axial compression relatively, and a lower passive confinement effect was observed. In addition, internal microcracks generation and merging processes were more rapid in high-strength concrete than those in normal concrete, which leads to a faster damage evolution.

### 4.3. Proposed Damage Evolution Equation

**Figure 8:**
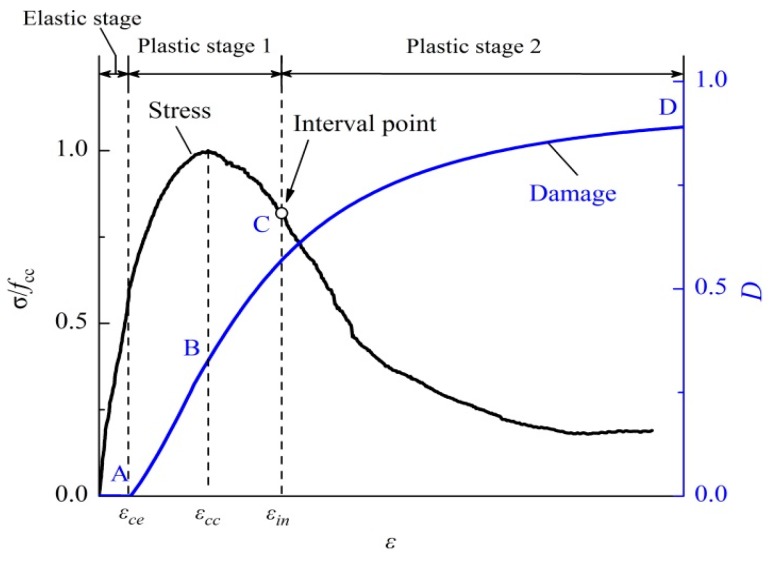
Characteristics of the damage evolution curve showing the different stages (Elastic, Plastic 1, and Plastic 2) related to the stress (**left** axis) and damage (**right** axis).

Figure 8 shows the damage evolution curve of a typical stirrup-confined concrete specimen (C1S2Y1, blue line) with stress–strain curve of monotonic compression (black line) superimposed. The test results revealed that the shapes of stress-strain curves of stirrup-confined concrete specimens were different from those of the unconfined concrete specimens. The former curves were broader, with an interval point located on the gradually declining branch, which was less steep than the latter curves (*d*^2^σ/*d*ε^2^ = 0 at the interval point). Based on the strain at interval point ε*_in_*, the plastic phase could be divided into two stages: plastic stage 1 and plastic stage 2. The damage evolution also presented distinct features in the different states:

1. In the elastic stage (Curve OA in Figure 8), no damage appeared, meant *D* was very small (close or equal to zero).
2. Plastic stage 1 included the ascending branch before peak stress and the declining branch after peak stress up to interval point. In the ascending branch, microcracks formed quickly, which resulted in rapid damage evolution, although with a small amount of damage (Curve AB in Figure 8). After peak stress, the crack merging processes progressed further, and damage accumulated during this stage. However, damage began to stabilize because of stirrup confinement effect (Curve BC in Figure 8).
3. In plastic stage 2, few new microcracks were generated, and damage increased very slowly while loading increased continuously until failure occurred (Curve CD in Figure 8).

The evolution of damage indicator of stirrup-confined concrete (*D_cc_*) is modified from *D_c_* by multiplying a confinement factor (*C*) as follows

(5) $$D_{cc}=\left\{ \begin{array}{l} 0\;\;\;\forall \varepsilon \leq \varepsilon_{ce}\\ C\times D_{c}\;\forall \varepsilon >\varepsilon_{ce} \end{array}\right.$$

where *D_c_* is the damage indicator of unconfined concrete defined by Li and Li [54], which is given by

(6) $$D_{c}=1-\frac{1}{1+{\left(\frac{Y_{c}-a_{c}}{b_{c}}\right)}^{l_{c}}}$$

where *Y_c_* is damage energy release rate (Li and Li [54]), which is given by

(7) $$Y_{c}=\left(1+k_{c}\frac{\varepsilon_{p}}{\varepsilon }\right)\times \frac{E_{0}\varepsilon_{ce}^{2}}{2}$$

In Equations (6) and (7), *a_c_*, *b_c_*, *l_c_* and *k_c_* are parameters of unconfined concrete (Li and Li [54]) that need be calibrated with experimental data and their values are summarized in Table 7.

Considering the different features of damage evolution before and after the interval point mentioned above, the expression of confinement factor (*C*) is given by

(8) $$C=\left\{ \begin{array}{l} \exp\left[m\left(-3.150-n\times \sqrt{\varepsilon }\times \ln(\varepsilon )\right)\right]\;\;\;\;\forall \varepsilon \leq \varepsilon_{in}\\ \arctan\left(A_{m}(\varepsilon -\varepsilon_{in})\right)\times 2(1-C_{in})/\pi +C_{in}\;\;\forall \varepsilon >\varepsilon_{in} \end{array}\right.$$

where *C_in_* is confinement factor corresponding to ε*_in_* × *C_in_* = 0.5 is suggested through the calibration with test data. The parameters *m*, *n*, and *A_m_* in Equation (8), which are obtained by the regression analysis of experimental results, are given by:

(9) $$m=0.06319f_{cc}{}^{1.1}\times {\left(1.296-\lambda_{v}{}^{0.7}\right)}^{2}$$

(10) $$n=0.53494f_{cc}{}^{0.15}\times f_{y}{}^{0.15}(5-1.79\lambda_{v}{}^{0.2})$$

(11) $$A_{m}=\exp(5.3+0.1\times f_{cc}{}^{0.4}/\lambda_{v}{}^{0.6})$$

**Figure 9:**
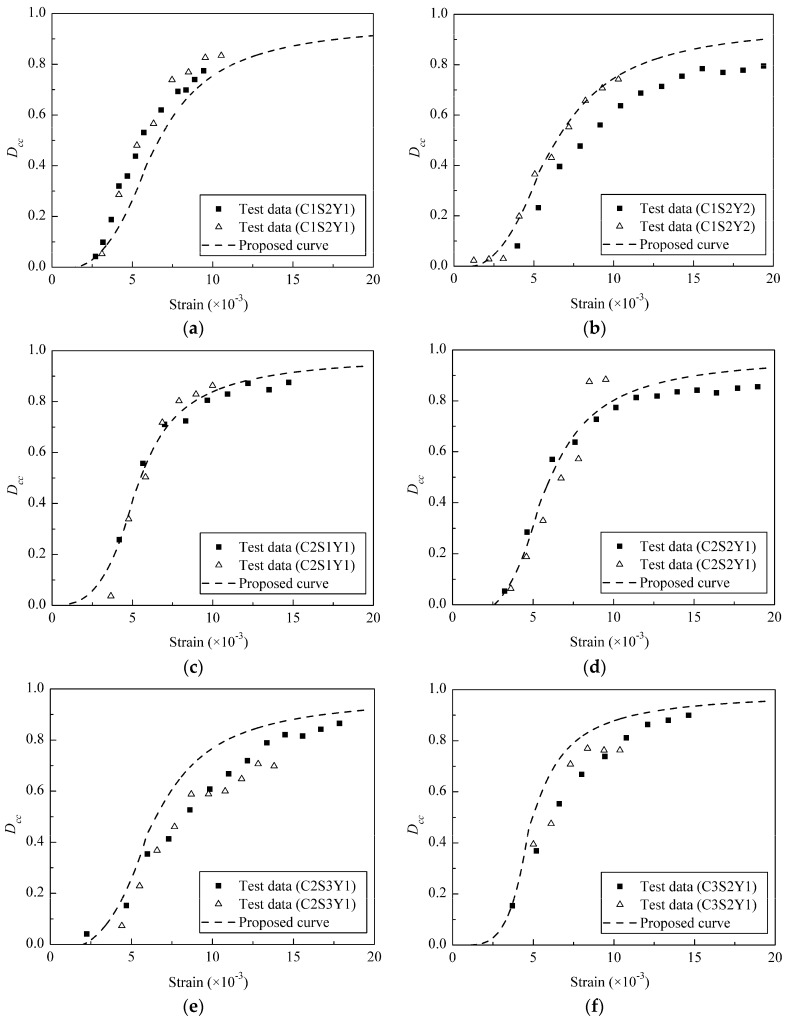
Comparisons of the proposed *D*_cc_ to the experimental results of specimens: (**a**) C1S2Y1; (**b**) C1S2Y2; (**c**) C2S1Y1; (**d**) C2S2Y1; (**e**) C2S3Y1; and (**f**) C3S2Y1.

The comparisons of proposed damage evolution with experimental results of the three stirrup-confined concrete specimen groups C1, C2 and C3 are shown in Figure 9. The damage began to increase from zero at a strain range of 2.0 × 10^−3^–4.0 × 10^−3^, displaying the same plastic deformation developments as those of the experimental data. The damage increased significantly before eventually stabilizing at *D_cc_* = 0.8–0.9, corresponding to the ultimate strain. In most cases, the proposed damage evolution curve accurately simulates the experiment results. It can be used to develop plastic damage models of stirrup-confined concrete.

## 5. Conclusions

A study of damage evolution of circular stirrup-confined concrete specimens under monotonic and cyclic compression loadings was presented. The effects of stirrup volume ratio, stirrup yield strength and concrete strength on stress-strain curves and damage evolution of stirrup-confined concrete were investigated. Based on experimental results, expressions for plastic deformation and damage evolution were proposed. The following conclusions can be drawn from this study:

- Strength and ductility of reinforced concrete can be improved by stirrup confinement effect. The ratio *f_cc_*/*f_c_*_0_ was introduced to describe the concrete strength improvement, which ranged from 1.07 to 1.19; additionally, the peak strain and ultimate strain displayed remarkable enhancements, as ε*_cc_*/ε*_c_*_0_ and ε*_ccu_*/ε*_cu_* were in the range of 1.43–2.12 and 1.70–2.79, respectively.
- The stirrup-confined concrete specimens showed clear transverse expansion instead of brittle failure, and the stirrups bowed out when failure occurred. The stirrups with lower stirrup volume ratios show limited confinement effect. At higher stirrup volume ratios, the stirrups provide higher confinement effect. Thus, the stirrup volume ratios play an important role in transverse confining of concrete.
- Confining pressure from stirrups reduces microcrack formation and restrained the damage evolution of concrete. As stirrup volume ratio increases, the stirrups provide a stronger transverse confining pressure, further restraining the damage evolution of concrete. Higher stirrup yield strength can generate a larger confining pressure, which would inhibit damage evolution. Due to the brittleness of high-strength concrete, growth and merging of microcracks proceed rapidly, causing the acceleration of damage evolution.
- Based on experimental results, a plastic strain expression was proposed, and a confinement factor (*C*) was introduced to the proposed damage evolution equation, to describe the effects of various confinement parameters on concrete damage evolution. The established damage evolution model can well represent the whole damage evolution process in circular stirrup-confined concrete. Because of less confinement parameters involved, the model can be conveniently applied to evaluate the plastic damage behavior of circular stirrup-confined concrete with reasonable accuracy.
- Evidently, more test results are needed to fully validate the proposed model. In addition, the size effect of specimen on damage evolution model was not considered in the present study. As such, the proposed damage evolution model for circular stirrup-confined concrete can be refined in future research works.

## Figures and Tables

**Table 1:** Material compositions of concrete.

Group	C1	C2	C3
Water (kg/m^3^)	185	185	195
Cement (kg/m^3^)	285	310	410
Gravel (kg/m^3^)	1145	1125	1055
Sand (kg/m^3^)	785	780	740

**Table 2:** 28-day cubic compressive strength of concrete (MPa).

Test	Group		
	A	B	C
1	21.2	25.1	38.9
2	22.0	24.8	40.0
3	20.7	26.5	42.6
Average	21.3	25.5	40.5

**Table 3:** Yield tensile strength (ultimate tensile strength) of stirrups and longitudinal steel bars (MPa).

Test	Y1	Y2	Longitudinal Steel Bar
	*d* = 6.0 mm	*d* = 6.5 mm	*d* = 10.0 mm
1	372 (518)	553 (652)	393 (530)
2	368 (522)	518 (672)	385 (529)
3	370 (520)	538 (669)	390 (532)
Average	370 (520)	536 (664)	389 (530)

**Table 4:** Mechanical and geometrical properties of all specimens.

Group	Specimen	*f_cu_* (MPa)	*d* (mm)	*s* (mm)	ρ_v_ (%)	*f_y_* (MPa)	λ*_v_*	ω*_wd_*	Number of Specimens
C1	C1S0Y0	21.3	–	–	–	–	–	–	4
C1	C1S2Y1	21.3	6.5	70	1.65	370	0.29	0.29	3
C1	C1S2Y2	21.3	6	70	1.65	536	0.42	0.42	3
C2	C2S0Y0	25.5	–	–	–	–	–	–	4
C2	C2S1Y1	25.5	6.5	140	0.82	370	0.12	0.12	3
C2	C2S2Y1	25.5	6.5	70	1.65	370	0.24	0.24	3
C2	C2S3Y1	25.5	6.5	46.7	2.47	370	0.36	0.36	3
C3	C3S0Y0	40.5	–	–	–	–	–	–	4
C3	C3S2Y1	40.5	6.5	70	1.65	370	0.15	0.15	3

**Table 5:** Test results.

Group	Specimen	*f_c_*_0_ or *f_cc_* (MPa)	ε*_c_*_0_ or ε*_cc_* (10^−3^)	ε*_cu_* or ε*_ccu_* (10^−3^)	*f_cc_*/*f_c_*_0_	ε*_cc_*/ε*_c_*_0_	ε*_ccu_*/ε*_cu_*
C1	C1S0Y0	18.69	2.09	4.02	–	–	–
C1	C1S2Y1	19.92	2.98	6.85	1.07	1.43	1.70
C1	C1S2Y2	20.23	3.1	7.27	1.08	1.48	1.81
C2	C2S0Y0	21.5	1.77	3.14	–	–	–
C2	C2S1Y1	22.92	2.56	5.92	1.07	1.45	1.89
C2	C2S2Y1	24.57	3.09	6.88	1.14	1.75	2.19
C2	C2S3Y1	25.59	3.76	8.76	1.19	2.12	2.79
C3	C3S0Y0	32.35	1.97	2.83	–	–	–
C3	C3S2Y1	35.8	2.87	5.59	1.11	1.46	1.98

**Table 6:** Existing models to predict *f_cc_/f_c_*_0_ and ε*_cc_*/ε*_c_*_0_.

Model	Peak Strength	Peak Strain	*f_cc_*/*f_c_*_0_	ε*_cc_*/ε*_c_*_0_
Kent and Park [3]	$\frac{f_{cc}}{f_{c0}}=1+\frac{\rho_{s}f_{y}}{f_{c0}}$	$\frac{\varepsilon_{cc}}{\varepsilon_{c0}}=1+\frac{\rho_{s}f_{y}}{f_{c0}}$	1.14−1.47	1.14−1.47
Mander *et al.* [23]	$\frac{f_{cc}}{f_{c0}}=2.254\sqrt{1+7.94\frac{f_{le}}{f_{c0}}}-2\frac{f_{le}}{f_{c0}}-1.254$	$\frac{\varepsilon_{cc}}{\varepsilon_{c0}}=1+5\left(\frac{f_{cc}}{f_{c0}}-1\right)$	1.11−1.84	1.54−5.21
Saatcioglu and Razvi [24]	$\frac{f_{cc}}{f_{c0}}=1+6.7\frac{f_{le}^{0.83}}{f_{c0}}$	$\frac{\varepsilon_{cc}}{\varepsilon_{c0}}=1+5\frac{k_{1}f_{le}}{f_{c0}}$	1.12−1.3	1.61−2.51
Present tests	–	–	1.07−1.19	1.43−2.12

**Table 7:** The parameter of *a_c_*, *b_c_*, *l_c_* and *k_c_* used to calculate damage.

Group	*a_c_*	*b_c_*	*l_c_*	*k_c_*
C1	0.0113	−0.191	1.0	−2.719
C2	0.0110	−0.432	1.0	−7.071
C3	0.0227	−1.579	1.0	−1.579

## Abbreviations

The following abbreviations are used in this manuscript:

IoT	Internet of Things
NDT	Non Destructive Testing
LVDT	Linear Variable Differential Transformer

## List of Symbols

*d*	Stirrup diameter
*s*	Stirrup spacing
ρ_v_	Stirrup volume ratio
λ*_v_*	Stirrup characteristic value
*f_y_*	Stirrup yield strength
*f_cu_*	Cubic compressive strength of the concrete
*F*_0_	Ultimate load of the unconfined concrete
*F*_c_	Ultimate load of the stirrup-confined concrete
*E*_0_	Elastic modulus of the unconfined concrete
*E_c_*	Elastic modulus of the confined concrete
*E_u_*	Secant stiffness of the line connecting present unloading point to next loading point
*f*_*c*0_	Peak compressive stress of the unconfined concrete
*f_cc_*	Peak compressive stress of the stirrup-confined concrete
ε	Strain
Δε	Strain increment
ε_*c*0_	Peak compressive strain corresponding to *f*_*c*0_
ε*_cc_*	Peak compressive strain corresponding to *f_cc_*
ε*_cu_*	Ultimate compressive strain of the unconfined concrete
ε*_ccu_*	Ultimate compressive strain of the stirrup-confined concrete
ε*_ce_*	Elastic compressive limit strain of the stirrup-confined concrete
ε*_p_*	Plastic strain
ε*_in_*	Plastic strain at the interval point
*D_c_*	Damage indicator of unconfined concrete
*D_cc_*	Damage indicator of stirrup-confined concrete
*C*	Confinement factor
*C_in_*	Confinement factor corresponding to ε*_in_*
*Y_c_*	Damage energy release rate function
